# Malignancy and IFITM3: Friend or Foe?

**DOI:** 10.3389/fonc.2020.593245

**Published:** 2020-12-08

**Authors:** Ushani S. Rajapaksa, Chen Jin, Tao Dong

**Affiliations:** ^1^ MRC Human Immunology Unit, MRC Weatherall Institute of Molecular Medicine, University of Oxford, Oxford, United Kingdom; ^2^ Chinese Academy of Medical Science Oxford Institute (COI), Nuffield Department of Medicine, University of Oxford, Oxford, United Kingdom; ^3^ Department of Liver Surgery and Liver Transplantation, West China Hospital, Sichuan University, Chengdu, China

**Keywords:** interferon induced transmembrane protein (IFITM3), interferon, transforming growth factor-β (TGF-β), epithelial to mesenchymal transition (EMT), tumor microenvironment (TME)

## Abstract

The prevalence and incidence of cancers has risen over the last decade. Available treatments have improved outcomes, yet mortality and morbidity remain high, creating an urgent demand for personalized and new therapy targets. Interferon induced transmembrane protein (IFITM3) is highly expressed in cancers and is a marker of poor prognosis. In this review, we discuss recent advances in IFITM3 biology, the regulatory pathways, and its function within cancer as part of immunity and maintaining stemness. Overexpression of IFITM3 is likely an indirect effect of ongoing inflammation, immune and cancer epithelial-to-mesenchymal (EMT) related pathways i.e., interferons, TGF-β, WNT/β-catenin, etc. However, IFITM3 also influences tumorigenic phenotypes, such as cell proliferation, migration and invasion. Furthermore, IFITM3 plays a key role in cancer growth and maintenance. Silencing of IFITM3 reduces these phenotypes. Therefore, targeting of IFITM3 will likely have implications for potential cancer therapies.

## Introduction

Cancer was the second leading cause of mortality in 2018, resulting in 9.6 million deaths globally (1). Cancer origins are multifactorial with genetic, and environmental contributions such as diet, UV, drugs, pollutants, smoking and others. Cancer occurs most commonly in tissues such as the lungs, breast, colorectum, prostate and skin. With recent improved treatments, especially newer immunotherapies, cancer mortality has been reduced and survival has increased. Yet, a large number of new cases are diagnosed annually, demonstrating a better understanding of cancer pathology is imperative (1, 2).

Recent focuses in cancer research have been towards understanding the cell extrinsic mechanisms of the tumor microenvironment (TME) and towards exploiting these mechanisms to treat and gain tumor control. This is especially important with immunotherapy now becoming a routine part of cancer treatment and as combinatorial treatments are being explored. One pathway that has emerged from this research is interferon (IFN) and interferon related signaling. IFN is generally considered a pathway that stimulates the immune response, but recent evidence indicates IFN signaling can also lead to immunosuppression and assist tumor spreading (3, 4).

IFN signaling induces transcription of a variety of proteins that are critical for cellular activities. Interferon-induced transmembrane proteins (IFITMs) are one such family of small proteins that are evolutionally conserved across vertebrates and single cells (5, 6). The human IFITM family is comprised of five members, the immune related genes IFITM1, IFITM2, and IFITM3, IFITM5, and IFITM10 which have no known role in immunity. IFITM1, 2 and 3 are ubiquitously expressed while IFITM5 is specifically expressed on osteoblasts. The aim of this review is to study existing literature to better understand the role of IFITM3 in tumors and the TME and to identify possible oncogenic and/or immunogenic roles for these proteins.

**Figure 1:**
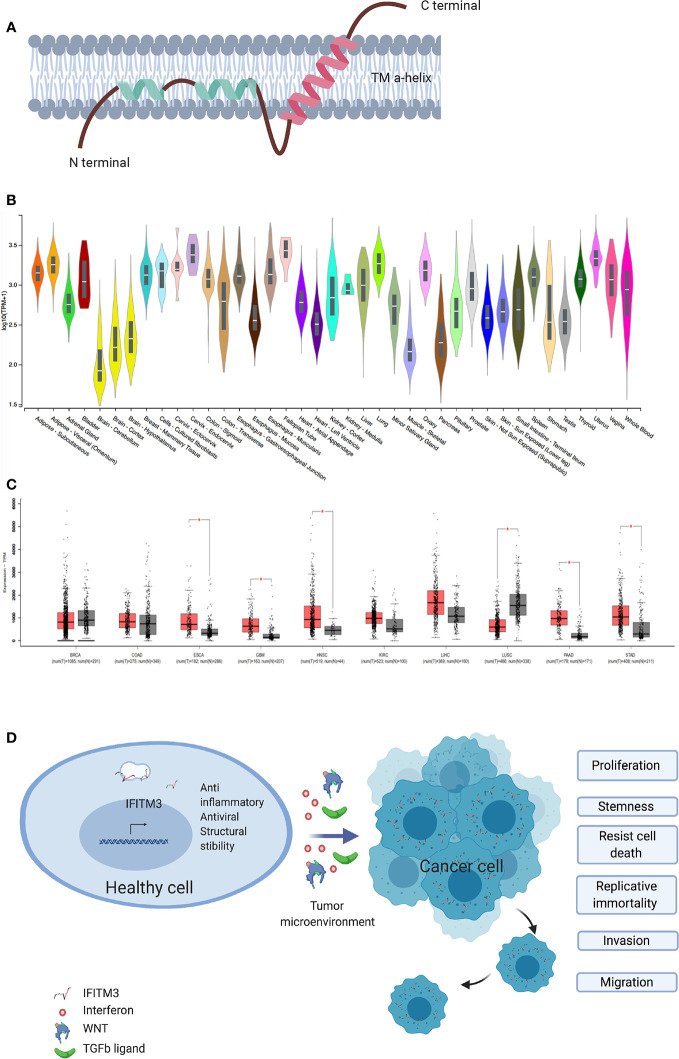
IFITM3 and malignancy. **(A)** The proposed structure of Interferon induced Transmembrane 3 protein. This consist of two transmembrane alpha helices with one each of hydrophobic and hydrophilic ends. Adapted from Ref (2). **(B)** IFITM3 is ubiquitously expressed in all tissues. Adapted from https://gtexportal.org/home/gene/ENSG00000142089. **(C)** Comparison of IFITM3 transcripts in common cancer types compared to normal tissue. Adapted from Ref (9). **(D)** Overexpression of IFITM3 positively influences all six hallmarks of cancer, namely proliferation, stemness, invasion, migration, uncontrolled division and resistance to apoptosis. BRCA, Breast invasive carcinoma; COAD, Colon adenocarcinoma; ESCA, Esophageal carcinoma; GBM, Glioblastoma multiforme; HNSC, Head and Neck squamous cell carcinoma; KIRC, Kidney renal clear cell carcinoma; LIHC, Liver hepatocellular carcinoma; LUSC, Lung squamous cell carcinoma; PAAD, Pancreatic adenocarcinoma; STAD, Stomach adenocarcinoma. Figure created with Biorender.com.

As key host defense genes, IFITMs evolved under the selective pressure of infections (7). IFITM3, also known as fragilis or I-8U is a 15-kDa protein encoded on human chromosome 11 and mouse chromosome 7 and is induced by type I, II and III IFNs (8). IFITM3 is a type 2 transmembrane protein (**Figure 1A**) (2) and has been intensely studied for its antiviral role in enveloped RNA viruses such as influenza, dengue, West Nile, HIV, and HCV (7, 10–12). Interestingly, the first description of IFITM3 comes from a genetic screen aimed at identifying the genes involved in the acquisition of germ cell competence (13, 14). Epiblast cells with the highest expression of IFITM3 initiated germ cell specification (15). IFITM3 is ubiquitously expressed in healthy tissues (16) and is usually located in late endosomes (2, 17). A number of IFITM3 single nucleotide polymorphisms (SNPs) and disease associations have been described. To date 13 synonymous, one in-frame stop and one splice site acceptor-altering SNPs have been reported in the translated sequence of IFITM3, with rs12552 perhaps being the best studied (10, 18). Hou et al. demonstrated an association between the rs12552 CC genotype and low differentiation, rapid progression, and higher relapse rate in hepatocellular cancer (19).

## IFITM3 Expression in Cancer

### The Relationship between IFITM3 Expression and Cancer

The role of IFITM3 in cancer is being increasingly scrutinized. Early work suggested IFITM3 is a cancer biomarker, following observed overexpression in colonic cancers and gliomas (20, 21). Subsequently, IFITM3 overexpression in cancer tissues as compared to healthy adjacent tissue was confirmed in other cancers, including colonic (22, 23), gastric (24), breast (25), prostate (26), lung (27), and liver (28) (**Figures 1B, C**). IFITM3 expression is higher in metastatic lymph nodes and bone metastases when compared to primary tumors (23, 26). In addition, overexpression of IFITM3 is also observed in precancerous conditions such as ulcerative colitis (29, 30). IFITM3 overexpression in inflammation is expected, given that IFN is a central mediator of inflammatory processes. However, the recent observation of IFITM3 overexpression in acute myeloid leukemia, a non-solid cancer, supports the hypothesis that IFITM3 overexpression is a hallmark of cancers generally and not just inflammation (31). The role of IFITM3 in cancer needs reassessing, as recent evidence suggests IFITM3 is one of the first genes activated in mouse colon cancer models (32) and precancerous colonic adenomas (20). However, the clinical significance and underlying mechanisms of dysregulated IFITM3 expression in cancers are still not well defined.

The location of IFITM3 in the cell interior may present important clues to IFITM3’s mechanisms of action. While IFITM3 localization in cancer cells has not yet been well described, IFITM3 appears to be localized both in the cytosol and the nucleus (28). Borghesan et al. demonstrated that IFITM3 also concentrated in small extracellular vesicles released from cells undergoing senescence, but not in intracellular multivesicular bodies (33). Studies into whether IFITM3 upregulation is seen only in transformed cancer cells or also in non-transformed stromal cells and whether IFITM3 is associated with a change in co-localization between cancer and normal healthy cells and stromal cells will likely provide important clues to IFITM3’s role in these cancer processes.

IFITM3 appears to also play a crucial role in cancer cell division and migration (**Figure 1D**). Many studies exploring the role of IFITM3 have used shRNA, siRNA knock out in cell culture systems and animal models. In gastric carcinoma cell lines, knockdown of IFITM3 significantly suppresses tumor cell migration, invasion and proliferation in vitro (24). Similar results are observed in colonic (23), breast (25), prostate (26), liver (28), glioma (21), and lung cancer cell lines (27). IFITM3 knock out tumor cells (IFITM3^-/-^) arrest at the G0/G1 phase and many cancer cell types display a reduced number of cells in the S phase (21, 23–25, 28). Reduced viability is observed in IFITM3^-/-^ breast and oral cancer cell lines (25, 34). Furthermore, IFITM3 overexpression increases cell proliferation, migration and invasion, all of which are hallmarks of cancer (**Figure 1D**).

Therefore, it is important to understand how IFITM3 expression is dysregulated in cancer. A reasonable inference is that IFITM3 upregulation is secondary to IFN in the TME. The fact that IFNs have anti-proliferation, anti-migration and anti-invasion properties is in contrast to what is seen when IFITM3 is overexpressed (35). This recurring pattern supports a tumorigenic role for IFITM3 (25) rather than an anti-inflammatory role and necessitates further examination.

### IFITM3 and Poor Prognosis

IFITM3 is a poor prognostic factor in colonic cancer and an independent risk factor for disease-free interval (23). Similar results are seen in acute myeloid leukemia (31), head and neck squamous cell cancer (36), and B-cell malignancies (37, 38). IFITM3 expression is positively correlated with cancer stage and differentiation status with higher expression levels in invasive ductal carcinomas as compared to ductal carcinoma in-situs (25) and non-differentiated lung cancers as compared to well-differentiated lung cancers (27). Additionally, IFITM3 is a negative prognostic marker in treatment outcome in esophageal (39) and hepatocellular cancer (28, 40), but, interestingly, not in glioblastomas (41). Conversely, another report suggests IFITM3 plays a crucial role in paracrine senescence *via* small extracellular vesicles, which are important in cancer treatment (33). Together, this raises the following questions: (1) whether IFITM3 overexpression is secondary to high/constant immune activation in the TME, (2) whether IFITM3 overexpression is inadvertently driving oncogenic properties, such as uncontrolled cell division, migration, and invasion—leading to poor prognosis and (3) how IFITM3 overexpression affects treatment outcomes.

### IFITM3 Promotes Cancer Metastasis

**Figure 2:**
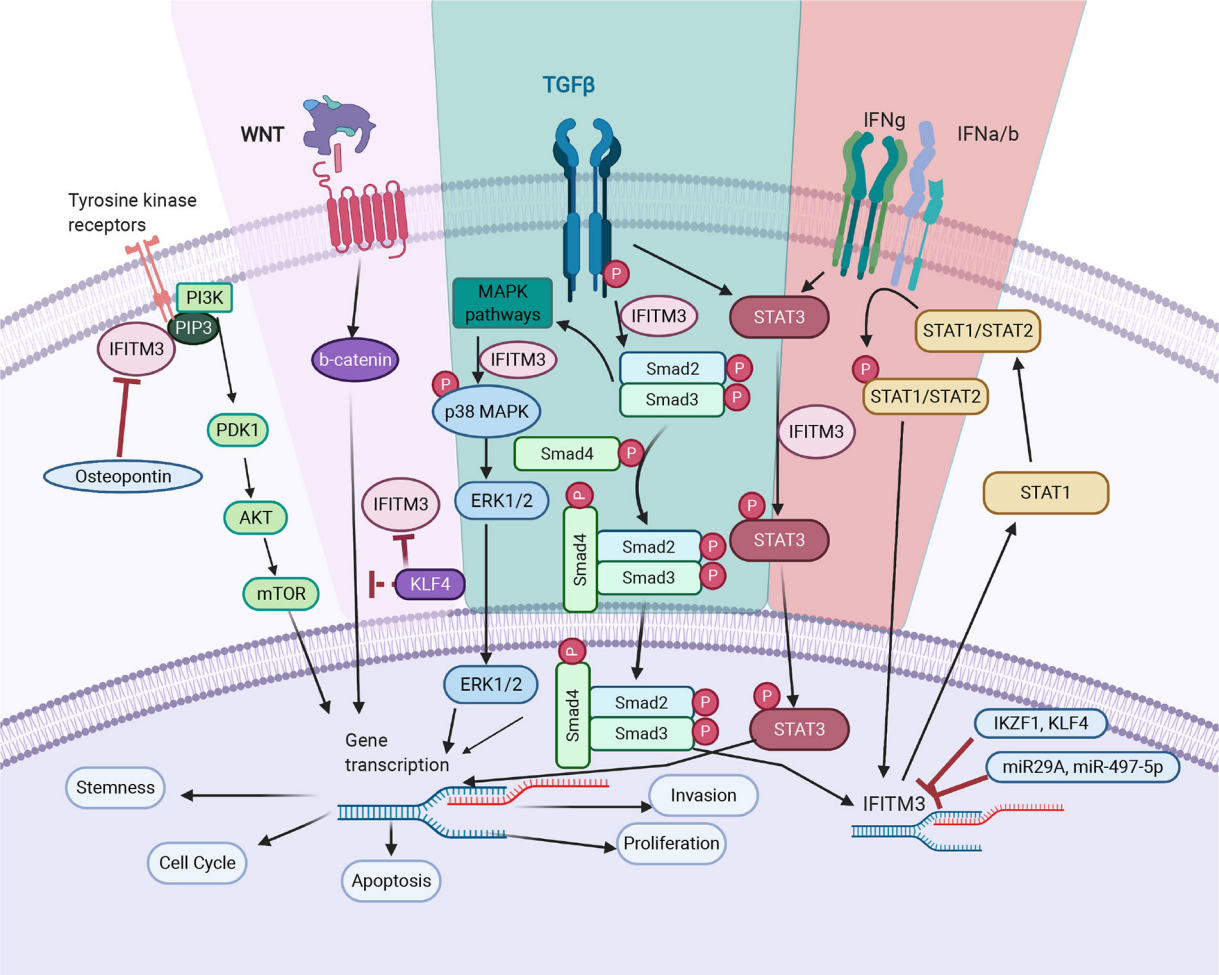
IFITM3’s mode of action in cancer. TGF- β pathway: IFITM3 stabilizes SMAD4 and SMAD2/3 phosphorylation and increases transcription of downstream oncogenic proteins. In non-canonical TGF-β signaling pathways, IFITM3 is involved in STAT3 signaling and activation of the p38/MAPK pathway, resulting in transcription of downstream oncogenic genes. JAK/STAT pathway: Upon activation of the JAK/STAT pathway following IFN, IFITM3 expression increases and interacts with other protein partners to increase transcription of downstream oncogenic and anti-inflammatory genes. PI_3_K pathway: IFITM3 interacts with PIP3 and PI_3_K, modulating PI_3_K/Akt signaling. Wnt pathway: IFITM3 levels are regulated by β-catenin, secondary to *APC* gene activation. In addition, KLF4 mediates *IFITM3* gene expression *via* both direct transcriptional inhibition and through attenuating Wnt/β-catenin signaling. The overall outcome of IFITM3 involvement in these various pathways is increased cell growth and proliferation, invasion, and metastasis. Figure created with Biorender.com.

Cancer metastasis is a leading cause of treatment failure and poor prognosis. It is a multistage process that involves proteolysis, migration of cells to adjacent and new tissues, cell division and neovascularization. Epithelial to mesenchymal transition (EMT) is central to metastasis and is triggered by a variety of autocrine and paracrine signals, as well as the blocking of some homeostatic mechanisms (42). IFITM3 plays a significant role in the metastasis of many tumors by regulating metastatic mechanisms. Blocking IFTIM3 reduces migration and invasion in a number of cancer cell lines (21, 25, 34, 40, 43). Indeed, transient overexpression increases tumor cell migration and invasion—supporting a role for IFITM3 in metastasis (28). In mouse models, wild-type cells readily establish metastases, as compared to IFITM3^-/-^ cells (26). In this section, we summarize some of the mechanisms by which IFITM3 is responsible for metastasis (**Figure 2**).

IFITM3 is a central component of a multi-protein complex involving the Src/FAK pathway. It assists in subcellular trafficking of phosphorylated Src and FAK between focal adhesion points—increasing cancer cell invasive properties (44). IFITM3 also promotes cholesterol aggregates in lipid rafts (45). These aggregates enable oncogenic signaling in B-cell malignancies by providing a robust membrane scaffold for tyrosine kinase (BCR-ABL1) and RAS-pathway oncogenes (*NRAS* ^G12D^) through PI(3,4,5)P3 increasing Src kinase and PI3K signaling (37, 46). The CD225 domain of IFITM3 is responsible for Src kinase/PI3K signalling (47).

Metalloproteinases, through their inherent ability to digest collagen, help numerous cancers progress by disrupting basal membrane integration (48). Metalloproteinases and IFITM3 are closely associated. IFITM3 knockdown reduces matrix metallopeptidase 9 and 2 (MMP9 and MMP2) in hepatocellular and gastric cancer cell lines (24, 26, 28). IFITM3 regulates MMP9 through p38/MAPK pathway activation (28). IFITM3 overexpression increases phosphorylated p38 in hepatic and prostate cancer cells (26, 28). IFITM3 overexpression also increases MMP9, ELK1, and STAT1 expressions. Activation of the p38/MAPK pathway is thought to result in a post-transcriptional mechanism that sustains a pro-tumorigenic effect (49). Zinc metallopeptidase, ZMPSTE24, which processes lamin A on the inner nuclear membrane and clears clogged translocons on the endoplasmic reticulum, also binds IFITM3 (50) and likely plays a role in this process; however, mechanistic details are still lacking.

MMP9 also promotes tumor angiogenesis (51). Since overexpression of IFITM3 drives MMP2 and MMP9 expression, IFITM3 overexpression also likely plays an important yet probably indirect role in stimulating angiogenesis (52). IFITM1 is critical for angiogenesis (53); however, direct evidence for IFITM3 contributing to angiogenesis is still lacking. Notably, IFITM3 is upregulated as part of the ISG response to hypoxia, which is a common stimulant for angiogenesis (54).

EMT is a critical process during metastasis. Downregulation of E-cadherin is a hallmark of EMT and results in a significant decrease of intercellular adhesion that subsequently contributes to robust migration and invasion of cancer cells (55). In contrast, N-Cadherin levels are upregulated during EMT and increase cell adherence (56). Studies show IFITM3 expression is inversely correlated with E-cadherin expression and is positively correlated with N-cadherin levels (24, 26). The mechanism for this correlation is still not well defined, but E-cadherin transcription is significantly reduced in IFITM3^-/-^ cells, suggesting IFITM3 may regulate E-cadherin transcription (24). There is indirect evidence that IFITM3 is an intermediate of both the β-catenin and MAPK pathways (24). Increased Wnt signaling leads to dissociation and translocation of β-catenin to the nucleus and destabilization of E-cadherin on the cell surface. MMPs also support this cell dissociation (57)—with both processes being supported by IFITM3. 

The TGF-β pathway regulates N-cadherin levels and involvement of IFITM3 in TGF-β signaling also likely contributes (58–61). Vimentin, a mesenchymal marker is downregulated in IFITM3^-/-^ gastric cancer cells, suggesting a direct association between these proteins (24). In addition, IFITM3 overexpression increases transcription of Snail, a canonical EMT marker (26). IFITM3 overexpression also increases production of FGF2, which can elicit EMT in neighboring cells in a paracrine manner (26).

Several other metastasis-related oncogenes associate with IFITM3. A close association between IFITM3 and c-myc is observed in B-cell acute lymphoblastic leukemia (B-ALL) and hepatocellular cancer cell lines (43, 62). C-myc is associated with cell proliferation and commonly dysregulated in cancer (63, 64). C-myc converges many oncogenic pathways, including Wnt/β-catenin, STAT3, MEK/MAPK, and others, to increases transcription of genes involved in cell cycle, apoptosis, proliferation and stemness. Downregulation of IFITM3 leads to a reduction of c-myc (62) which is regulated through ERK1/2 in HCC (43). Details of this regulation mechanism and whether IFITM3 activates transcription or is a binding partner of c-myc is yet to be determined.

## Regulation of IFITM3 Expression

A clearer understanding of the regulatory mechanisms responsible for aberrant expression of IFITM3 may determine whether overexpression is secondary to immune activation, or to oncogenic mechanisms, and or whether immune-mediated upregulation inadvertently results in driving oncogenesis. Several regulatory factors associated with IFITM3 are discussed below (**Figure 2**).

### Interferons

IFNs are the main regulators of ISGs, of which IFITM3 is a part of, and induce expression in a hierarchically –dependent-manner based on cell and tissue type (8). IFN mediated activation occurs through IFN receptors in the JAK/STAT pathway and has been reviewed before (8). Notably, IFITM3 is likely part of a positive feedback loop where IFITM3 increases STAT1 transcription and STAT1 increases IFITM3 transcription (28). IFN stimulation increases transcription of IFITMs by 8- to 20-fold within 4 to 8 h and is dependent on the strength of the stimulus (65) IFNs are readily found in the TME and are secreted by cancer, immune and bystander cells. Therefore, it is possible that IFNs in the TME drive IFITM3 expression in cancer and stromal cells. Curiously, IFNs possess anti-tumor activity (66), contrary to the phenotype driven by downstream IFITM3 expression.

### Transforming Growth Factor-β

Transforming Growth Factor-β (TGF-β) plays a key role in EMT (67). Early in cancer development, TGF-β exerts an anti-oncogenic effect but later becomes pro-oncogenic (68). Canonical TGF-β signaling involves SMAD proteins and signaling through cofactors that lead to gene transcription. However, non-canonical TGF-β signaling involves the MAPK and PI3 pathways (69). The source of TGF-β is typically immune cells in the TME and occasionally cancer cells.

The relationship between TGF-β and IFITM3 appears complex. TGF-β stimulation increases IFITM3 transcription in prostate and glioblastoma cancer cell lines (26, 70). SMAD4 and TGF-β receptors are downregulated when IFITM3 is silenced and this is more pronounced in the presence of TGF-β (26). A close relationship between IFITM3 and SMAD4 was noted using co-immunoprecipitation methods. IFITM3 blocking led to suboptimal TGF-β signaling with reduced phosphorylation of ERK-1 and SMAD2 and reduced fibroblast growth factor 1, fibroblast growth factor 2 and parathyroid hormone-related peptide, all of which promote EMT and bone metastasis (26).

Emerging evidence also suggests involvement of IFITM3 in several non-canonical TGF-β signaling pathways. One such pathway is through the signal transducer and activator of transcription 3 (STAT3). Ex-vivo TGF-β stimulation increases STAT3 activation, which is important for invasion and metastasis of many cancers (58). Activated STAT3 increases c-myc, an oncogene responsible for EMT. Knocking down IFITM3 reduces TGF-β and leads to STAT3 phosphorylation, which is crucial for glioma development. This evidence supports IFITM3 as an important TGF-β pathway intermediate (70).

Studies of non-canonical TGF-β signaling pathways suggest MAPK and PI3K activation converge on SMAD signaling. Interestingly, IFITM3 is critical in all of the above pathways, making it an essential partner in TGF-β signaling.

### Wnt/β-Catenin

Wnt/β-catenin signaling is involved in many cellular processes (71). Wnt/β-catenin controls the maintenance of somatic stem cells in many tissues/organs and is implicated in carcinogenesis by regulating cell cycle progression, apoptosis, EMT, angiogenesis, stemness, and tumor-immune micro-environments (72, 73). In the canonical pathway, upon receptor stimulation, β-catenin translocates across the nuclear membrane and transcribes genes such as cyclin D1, cyclin E, MMP-7, c-myc, VEGF, and others (71). These genes are involved in various hallmarks of cancer. In addition, β-catenin serves as a major structural component of E-cadherin–mediated multiprotein complexes that maintain cell polarity and cell-cell adhesion. Many downstream targets of β-catenin are components of the ECM, including laminin, a key protein in the basement membrane, lysyl oxidase and fibronectin that reside in the interstitial matrix, as well as invasion-associated genes such as MMP-9, MMP-74, and CD44 ligands.

The first evidence for the cancer association of Wnt and IFITM3 comes from colonic cancer (74). IFITM3 levels were noted to be regulated by β-catenin, secondary to activation of the *APC* oncogene. Blocking β-catenin in gastric cancer cells using XAV939 lead to a reduction of IFITM3, supporting the above hypothesis (24). However, the mechanism by which IFITM3 is regulated is still not clear. Possibilities include, β- catenin regulating transcription of IFITM3 or that β-catenin is a structural component for stabilizing IFITM3 on cell membranes. Both possibilities require further studies.

Dawei Li et al. demonstrated that Kruppel Like Factor 4 (KLF4), which plays a critical role in colon cancer progression and metastasis, regulates IFITM3 transcription (23). KLF4 is closely related to the Wnt/β-catenin pathway, and directly interacts with TCF (75). In addition, expression of Wif1, an intermediate of Wnt signaling, and IFITM3 are increased in a colorectal cancer induction model—supporting a functional relationship between Wif1 and IFITM3 in cancer initiation (32). Thus, the interactions between IFITM3 and Wnt/β-catenin are likely complex and multi-dimensional (32).

WNT also creates a favorable environment for TGF-β induced EMT (76). Therefore, if IFITM3 is an intermediate in TGF-β signaling, IFITM3 may also unite these two cancer-related pathways.

### MiRNAs and Long Non-coding RNAs

MicroRNAs (miRNAs) are a class of endogenous non-coding RNA capable of post-transcriptionally regulating gene expression through repressing protein translation or silencing the expression of target genes. These processes play a critical role in various cancers. A small number of miRNAs associate with IFITM3. Liang et al. demonstrated that miRNA29a regulates IFITM3 (40). MiRNA29a is a protective factor in HCC, mediating its effect through SPARC, CLDN1, and TGF-β, and is a direct negative regulator of IFITM3. Both of which are central to exerting miRNA29a’s protective effect, although details of downstream mechanism are yet to be confirmed (40). Similarly, miR-497-5p, a tumor-suppressor, is inversely correlated with IFITM3 in pancreatic cancers, suggesting IFITM3 is a downstream target (77).

### Other IFITM3-related Pathways

Many cellular pathways regulate IFITM3, but the finer details are not well understood. KLF4 is one such transcription factor and a tumor suppressor that is linked to several cellular pathways (78). Aberrant KLF4 is associated with overexpression of IFITM3 in colon cancer (23). KLF4 mediates IFITM3 expression *via* both direct transcriptional inhibition and attenuation of Wnt/β-catenin signaling. KLF4 downregulates IFITM3 transcription *via* two putative IFITM3 promoter binding sites. KLF4 also directly interacts with the C-terminal transactivation domain of β-catenin and inhibits Wnt/β-catenin signaling in intestinal cancers (75).

It is also suggested that the transcription factors IKZF1/IKAROS repress IFITM3 and play a role in focal point adhesion (38). RNA binding proteins, G3BP1 and G3BP2, regulate IFITM3 expression, likely through the MEK/ELK pathway and also possibly through the of binding the 3′UTR of IFITM3 to increase protein expression (3).

Intriguingly, although part of an ISG signature, anti-cancer or anti-inflammatory roles are rarely attributed to IFITM3. An exception is a report where IFITM3 reduces mRNA expression of osteopontin (OPN) by directly binding the promoter, in the absence of p53 or other proteins. This occurred in a hybrid system and abolished the tumorigenic properties induced by OPN (79). OPN is an extracellular matrix glyco-phosphoprotein that binds to integrins and is important for malignant cell transformation, attachment and migration (80). Overexpression of OPN occurs in gastric cancers and is correlated with early metastasis and poor prognosis in breast and gastric cancers. In addition, a recent study described an anti-inflammatory role for OPN in colitis and associated tumorigenesis in a mouse model (81).

## IFITM3 and Stemness

Cancer stem cells have gained momentum in understanding cancer behavior. Cancer stem cells, with their unregulated, and primarily symmetric, cell division, result in tumor spreading and radiation resistance in many cancers (82).

An elegant paper from the Charles Rice group observed high ISG expression, including IFITM3, in induced pluripotent stem cells (83). Thought to be inherently expressed to protect cells from viral infections, IFITM3 levels went down with cell differentiation. IFITM1/3 expression was also higher in the basal/pluripotent layer of squamous cervical cell cancers but not in differentiated cells (84). This has two primary implications, either IFITM3 is important for establishing stemness and/or IFITM3 contributes to maintaining stemness. Unpublished data from our lab demonstrates tumor spheres are readily formed in WT cells compared to IFITM3^-/-^ cells. Indeed, inflammation in the TME leads to overexpression of ISG resulting in dysregulated immune responses, a known contributor to tumorigenesis (3, 85). In fact, an interferon related DNA damage signature (IRDS) is thought to result in poor tumor outcome by mechanisms that include EMT and consequently increased metastatic potential, suppression of T-cell toxicity, resistance to therapy – likely driven by tumor stem cells (85). It will be interesting to determine whether IFITM3 contributes to this process similar to its counterpart IFITM1 (86, 87), a result that may explain the association with metastasis and low differentiation.

## IFITM3 in Immunity

IFITM3 and immunity are closely related and play an important role in viral infections, specifically following IFN stimulation (88). The role of IFITM3 in tumor immunity is not well described but cellular distributions may clarify whether IFITM3 is tumorigenic, or merely part of a reaction to cytokines in the TME, and whether IFITM3 overexpression occurs only in transformed cancer cells or in stromal cells or both.

IFNs have a complex and dynamic role in cancer immune responses and treatment success (89). Activated T-cells and antigen presenting cells secret IFNs in response to cancer cells. Gomez-Herranze et al. show IFN-g induced Human Leukocyte Antigen-I expression depends on IFITM1/3, supporting a role for antigen presentation in cervical tumors (84). This effect was independent of b2M and STAT1. Although, the lack of a specific antibody to screen single gene knock-outs leads to difficulty in ascribing this effect directly to IFITM3, the finding has important implications. Increased HLA-I expression is inversely correlated with cancer progression (88, 90, 91). Shen et al. showed MHC-II transcription is downregulated in the absence of IFITM3 (92). While KIR, calreticulin, and DQ α2 are also downregulated, heat shock protein 90 and 70 are upregulated in IFITM3 knock out cells (92). Involvement of IFITM3 in these processes demonstrates its role in tumor associated antigen presentation and specific immune engagement for keeping tumor and immune system equilibrium.

IFITM3 is expressed in both CD4+ and CD8+ T-cells, yet its function appears complex (93). IFITM3 expression levels likely rise secondarily to IFN during tumor immune/inflammatory responses. It is not currently known how rich the TME is for IFNs. Bedford et al. noted that engagement of T-cell receptors is sufficient to upregulate IFITM3 and this is independent of IFN, STAT1 and IRF3 in both CD4+ and CD8+ T-cells (93). This situation likely occurs in cancers, as opposed to viral infections, where strong innate sensing is triggered. Interestingly, a lack of IFITM3 favors a shift toward Th1 (88), suggesting IFITM3 favors a Th2 shift (94). Th2 cells generally favor tumor growth by inhibiting cell-mediated immunity and by favoring angiogenesis (95). It is reasonable to postulate that overexpression of IFITM3 induces a Th2 shift that causes detrimental effects to the patient.

Lastly, a recent paper shows IFITM3 is correlated with the inhibitory immune checkpoint receptors PD-L1, B7-H4, VISTA, IDO, and the tumor associated macrophage markers CD68, CD163, and CD206, which are associated with tumor immunosuppression (36). However, the mechanistic details of this correlation are still lacking. IFITM3 negatively regulates inflammatory responses by accelerating IRF3 turnover in autophagosomes, thereby reducing protein levels and phosphorylation (96). It appears likely that IFITM3 overexpression subverts innate cytosolic sensing triggered *via* cGAS/IRF3 (97). This reduces transcription of type 1 IFNs, and reduces subsequent immune responses. IFITM3 also negatively regulates activation of NF-κB (96), a pro-tumorigenic protein (98). Furthermore, IFITM3 suppresses IL-6 production, a pro-inflammatory cytokine required for cancer cell migration and invasion (99, 100). Moving forward, many interesting avenues remain for pursuing IFITM3’s immunological and cancer roles.

## Conclusion

Recent studies have identified new roles for IFITM3 that are independent of its classical antiviral activities. As discussed here, IFITM3 has a multi-dimensional role and may join multiple signaling pathways that are responsible for oncogenesis and tumor progression. Direct overexpression of IFITM3 is associated with EMT, increased migration and invasion of tumor cells—yet a detailed mechanistic understanding for these phenotypes is still lacking. In addition, IFITM3’s role in shaping innate and adaptive immune responses demonstrates a wider role in the TME and in the fine balancing of tumor immune responses and inflammation. It seems reasonable to speculate that overexpression of IFITM3 is part of an anti-tumor immune/inflammatory response. Factors such as TGF- β, and IFNs, in the microenvironment, not only upregulate expression of IFITM3, but also contribute to upregulation of immunosuppressive molecules on both immune and cancer cells, facilitating immune escape. There is strong evidence from solid and hematological malignancies that IFITM3 itself has oncogenic properties and that these functions drive tumor progression. Therefore, the overall effect of high IFITM3 expression appears to be cancer progression and poor survival.

More research is needed to understand how IFITM3 expression changes in tumor and stromal cells as compared to normal cells, and how IFITM3 expression changes affect downstream signaling in these cells. This work will help clarify the above speculations. Additionally, identification of the domain(s) responsible for the tumorigenic properties of IFITM3 and whether these domain(s) differ from those required for immune responses will also help explain this mechanism.

There is still much work to be done for confirming the mechanisms by which IFITM3 affects cancer progression, including progression of hematological malignancies. The ability of IFITM3 to unite many signaling pathways has the potential to be applied in multitude of cancers. Blocking IFITM3 may lead to changes in downstream signaling and help abrogate tumor progression – an observation that has important implications for designing future cancer therapeutics. Moving forward, we expect to see IFITM3 playing a larger role in human cancer and disease studies, as well as signaling pathways and personalized cancer treatments.

## Author Contributions

UR, CJ, and TD were responsible for the conception and design of the study and writing, reviewing, and editing of the manuscript. All authors contributed to the article and approved the submitted version.

## Conflict of Interest

The authors declare that the research was conducted in the absence of any commercial or financial relationships that could be construed as a potential conflict of interest.
